# Case Report: Spontaneous acute hemopericardium

**DOI:** 10.3389/fcvm.2024.1414519

**Published:** 2024-10-01

**Authors:** Manuel Chacón-Diaz

**Affiliations:** Unidad Cardiovascular, Clínica Delgado AUNA, Lima, Peru

**Keywords:** hemopericardium, pericarditis, aortic diseases, cardiac tamponade, tuberculosis

## Abstract

Acute hemopericardium is generally produced by complications of interventional procedures or traumatisms to the chest wall. In absence of those antecedents, clinicians face an arduous process of etiological diagnosis and treatment. We present the case of a male patient with history of Hodgkin's lymphoma and aortic endovascular treatment years ago, who develop an episode of fever, chest pain and dyspnea that was complicated with cardiac tamponade diagnosed with echocardiogram and angio- tomography. In the operating room hemopericardium was diagnosed and drained with resolution of symptoms. Diagnosis work out was carried out with suspicion of tuberculous or neoplastic pericarditis with negative results. Patient was discharged with the diagnoses of viral or idiopathic pericarditis. The case highlights the use of multimodality images and laboratories procedures to lead to a correct diagnoses and treatment.

## 1 Introduction

Pericarditis is the inflammation of the pericardium due to various local or systemic, infectious, or non-infectious factors, and sometimes its etiology can remain unknown. It is the most common disease of the pericardium, accounting for approximately 5% of emergency department visits for chest pain in North America and Europe (1). Pericardial effusion may be present in up to 65% of cases of acute pericarditis and can lead to cardiac tamponade, especially in certain etiologies such as neoplastic or tuberculous (TB) pericarditis (2, 3).

Hemopericardium is the presence of a serosanguineous or frank bloody pericardial effusion, predominantly traumatic in its etiology, but also secondary to myocardial infarction, aortic pathology, neoplastic, or infectious diseases (4). In this report, we present the case of a patient with non-traumatic hemopericardium, emphasizing the diagnostic process in search of a specific etiology for treatment.

## 2 Case description

A 53-year-old male with a history of Hodgkin's lymphoma at age 17, who was treated with chemotherapy and radiotherapy with complete remission. One year ago, he underwent fenestrated endovascular repair of the thoracic aorta with chimneys to the right brachiocephalic trunk and subclavian artery due to aortic ulcers in the ascending aorta and aortic arch, and an intramural hematoma in the ascending aorta. He also had a history of mild aortic stenosis and type 2 diabetes mellitus. At admission, he was on medication with metformin 750 mg/sitagliptin 50 mg per day, aspirin 81 mg per day, clopidogrel 75 mg per day, rosuvastatin 20 mg per day, and bisoprolol 5 mg per day.

He was totally asymptomatic until 14 days before admission. Four days before symptom onset, he exerted physical effort (pushing his car a couple of meters) without reporting chest contusion. The disease began with fever, malaise, diarrheal episodes, and retrosternal pleuritic pain, progressing with dyspnea on moderate exertion that led him to the emergency room.

Initial evaluation revealed a patient tolerating a supine position, with blood pressure of 130/70 mmHg, heart rate of 110 bpm, respiratory rate of 15 bpm. Cardiovascular examination found jugular venous distension ++/++, rhythmic heart sounds, presence of a systolic ejection murmur at the aortic focus, and absence of paradoxical pulse.

**Figure 1:**
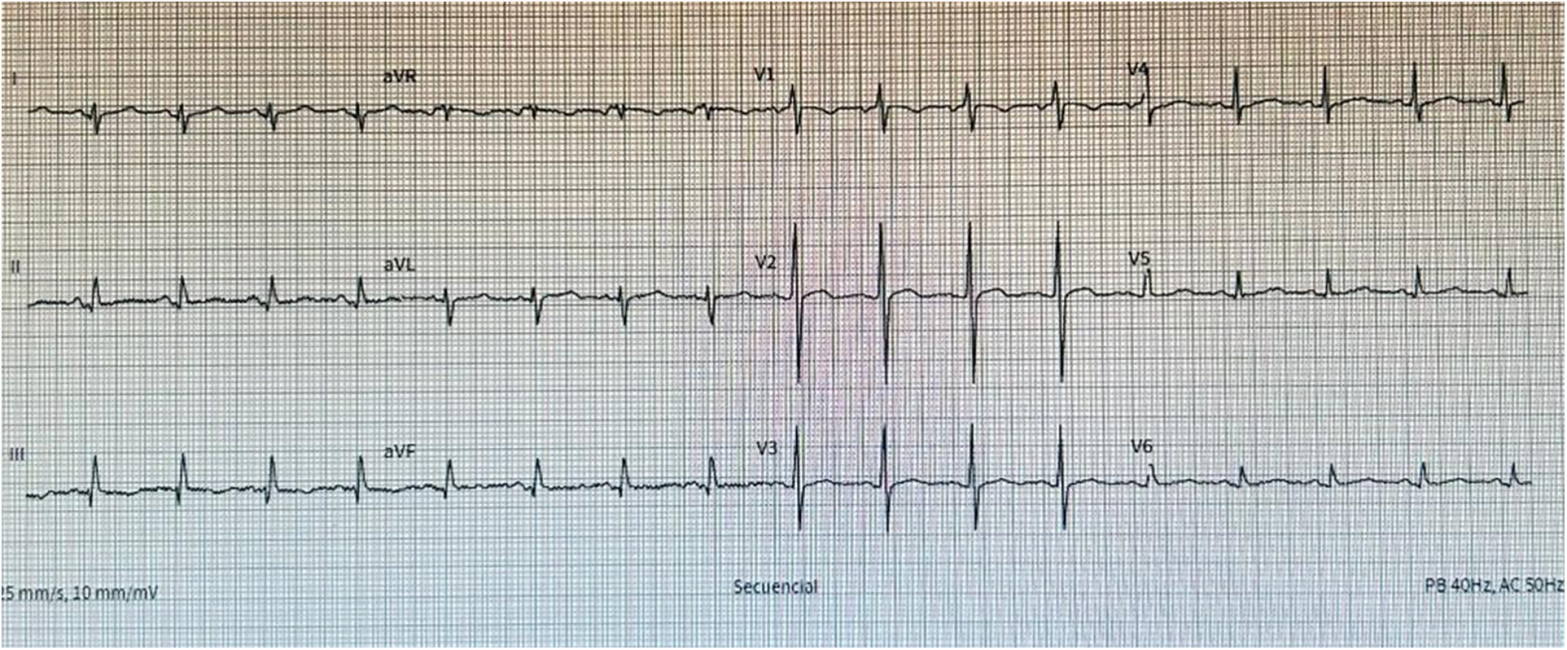
Electrocardiogram at emergency arrival showing sinus tachycardia, no signs of ischemia nor electrical alternans.

**Figure 2:**
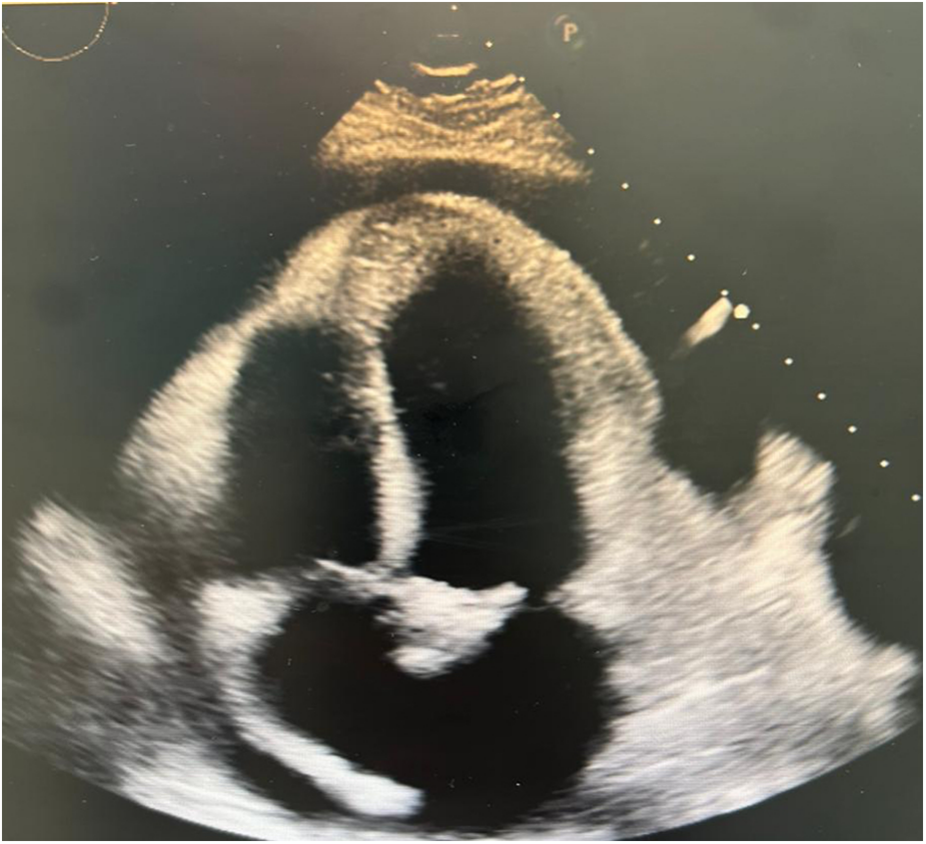
Echocardiogram 2D showing pericardial effusion, no signs of tamponade.

The electrocardiogram showed sinus tachycardia (Figure 1) and chest x-ray demonstrated mediastinal widening. Laboratory results are shown in Table 1. The echocardiographic study showed a left ventricular ejection fraction of 65%, mitral E wave velocity of 134 cm/s, and mitral A wave velocity of 121 cm/s. E/e': 20. Mild calcified aortic valve stenosis and severe pericardial effusion without signs of tamponade (Figure 2).

**Table 1:** Blood test results at admission.

Test	Results	Normal value
Leukocytes	8,940 cells/ul	5,000–10,000 cells/ul
Neutrophils	75%	–
Lymphocytes	11%	–
Hemoglobin	11.6 g/dl	–
Platelets	330,000 cell/ul	–
C-reactive protein	6.6 mg/dl	0–0.5 mg/dl
Procalcitonin	0.02 ng/ml	0–0.5 ng/ml
Pro-BNP	407 pg/ml	–
Troponin T	0.004 ng/ml	0–0.05 ng/ml
D-dimer	5.1 ug/ml	0–0.5 ug/ml
Ebstein Barr Ig M	4.4 U/ml	0–25
Cytomegalovirus IgM	0.5	0–1.1

**Figure 3:**
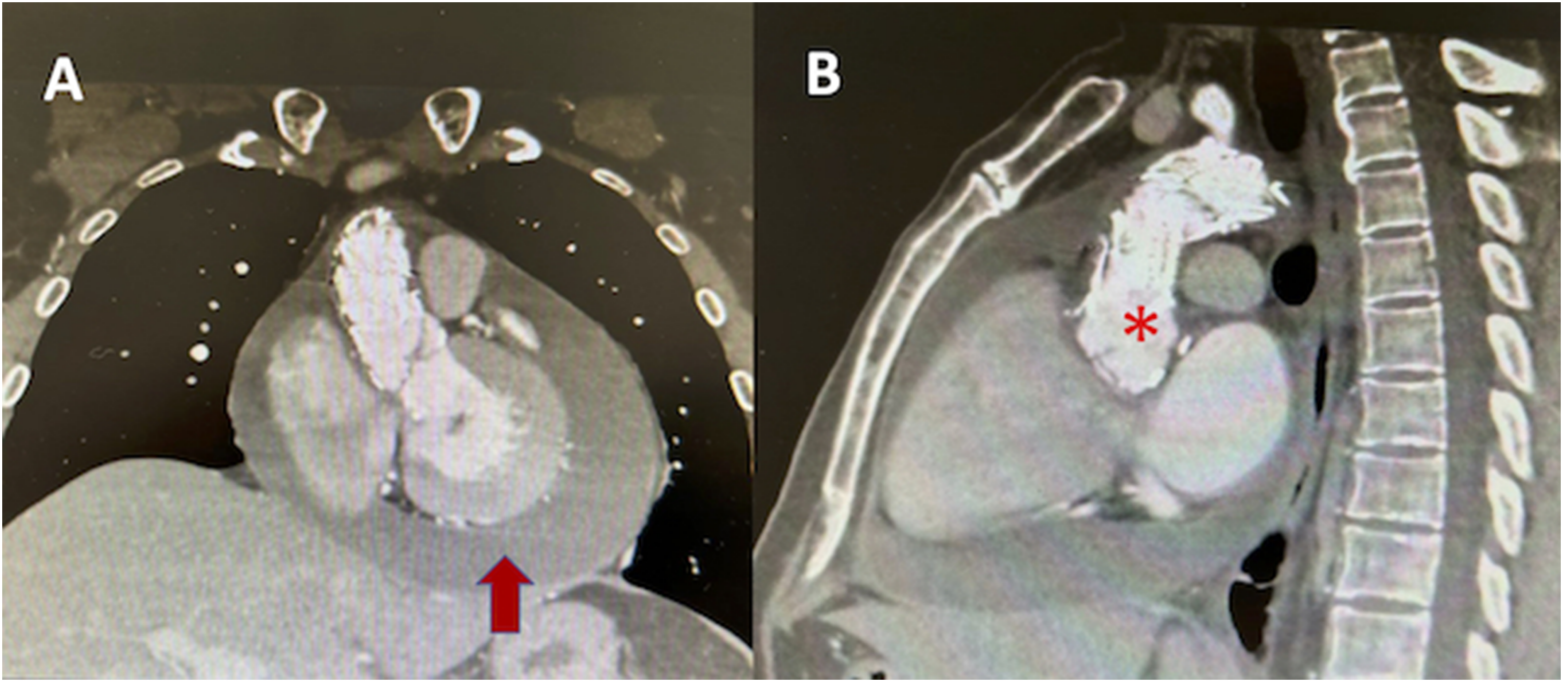
Angio CT of aorta **(A)** coronal axis showing pericardial effusion (red arrow), **(B)** endovascular material in ascending aorta (asterix).

Given the cardiovascular history, an aortic and great vessel's Angio CT was performed, revealing the aortic endoprosthesis in position, without leaks, dissection, or aneurysms (Figure 3). With the diagnosis of acute pericarditis and pericardial effusion, the patient was hospitalized and received oral aspirin and colchicine.

Film array test for respiratory viruses (COVID 19, Influenza, Parainfluenza, syncytial respiratory virus, Adenovirus, Herpes virus, Parvovirus, Coxsackie virus, etc.), elisa for HIV, viral hepatitis antigen, and Quantiferon for TB were all negatives.

The next day, clinical signs of cardiac tamponade (paradoxical pulse) developed, prompting surgical drainage of pericardial fluid. In the operating room, under general anesthesia, a pericardial window was performed with subsequent pericardial biopsy and pericardial fluid drainage (around 500 cc of serosanguineous fluid). Pericardial fluid analysis showed a glucose of 55 mg/dl, proteins of 4.6 g/dl, LDH of 1,728 U/L, cholesterol of 79 mg/dl, leukocytes 821 cells/μl, polymorphonuclear cells (18%), mononuclear cells (82%), more than100 red blood cells per field, presence of clot and no microorganisms. Gene X-pert for TB was negative. Adenosin deaminasa test (ADA) was 65.2 U/L (normal value <50 U/L).

Block cell analysis of pericardial fluid showed a fibrin-leukocyte clot without evidence of malignant neoplasia. Other test like carcinoembryonic antigen (CEA), lupus anticoagulant, LE cells, and antinuclear antibodies (ANA) were negative.

The patient's condition improved immediately after pericardial drainage, with the resolution of fever and cardiovascular symptoms. Given the positive ADA, specific anti-tuberculosis therapy was initiated, and the patient was discharged with specific treatment for TB and prednisone 60 mg QD four days later.

Subsequent biopsy results of the pericardium reported fibro-conjunctive tissue with chronic inflammation, hemorrhage, and granulation tissue. No granulomas or neoplastic infiltration were observed. We decided to discontinue anti-tuberculosis drugs after 3 weeks of treatment and continue colchicine for 3 months assuming an idiopathic spontaneous hemopericardium (possible viral).

## 3 Discussion

Acute pericarditis is characterized by pericardial inflammation with symptoms lasting 4–6 weeks (1). Diagnosis requires at least 2 of the following 4 criteria: chest pain, pericardial friction rub, electrocardiographic changes, or pericardial effusion, supported by laboratory or imaging evidence of inflammation (5). Typical electrocardiographic changes occur in 60% of cases and may not be evident in the presence of pericardial effusion.

Echocardiography may be normal in acute pericarditis but is essential to demonstrate pericardial effusion and to semi-quantitatively describe its size in diastole as trivial (systolic only), mild (<10 mm), moderate (10–20 mm), severe (21–25 mm), and very severe (>25 mm) (6). Additionally, it allows assessment of hemodynamic compromise and signs of cardiac tamponade through a Doppler study of mitral and tricuspid flow velocities (change of >30%–40% suggestive of tamponade) (7). In or case, echocardiography was crucial for diagnosing pericardial effusion and to initiate management, although the mentioned Doppler study was not performed.

Due to the past history of aortic pathology with vascular endoprosthesis, the initial step was to rule out acute aortic syndrome (aortic dissection) or endoprosthesis complications via aortic CT, as this would require emergency surgical treatment. Initial findings suggestive of inflammation without troponin elevation or ventricular motility compromise in echocardiography led to the exclusion of myopericarditis.

Acute hemopericardium is usually a complication of invasive procedures (catheterization, coronary angiography, arrhythmia ablation, pacemaker placement, etc.) and may account for up to 30% of those cases (4). However, it can also occur in the context of myocardial infarction, aortic dissection, and systemic pathologies such as neoplasms, autoimmune diseases, and, in our region, infections such as TB.

In this case, after ruling out acute aortic pathology and diagnosing hemopericardium, the initial suspicion was neoplastic involvement due to the history of lymphoma, as this entity frequently metastasizes to the pericardium (after lung, breast, and esophagus cancer) (8), however, this was ultimately ruled out by Block cell analysis and pericardial biopsy results. The history of chest radiation therapy in adolescence also raised suspicion of radiation-induced pericarditis, as the incidence of constrictive pericarditis is up to 7% in patients with such a history (9, 10). However, no evidence of effusive-constrictive pericarditis was found on imaging or clinical presentation after pericardial drainage.

Suspicion of TB pericarditis was based on the endemic area in which we live and the positivity of the ADA test in pericardial fluid, despite its limitations as a diagnostic test, requiring confirmation by histopathological examination (sensibility 10%–64%) and culture for TB (sensibility 53%–75%) (11), both negatives in this patient. Pericardial effusion in TB is generally a protein-rich exudate with abundant lymphocytes, and hemopericardium may be present in up to 80% of cases (12). However, as seen, it is not specific to this etiology.

Some viral infections, such as Coxsackie B virus, and parvovirus predominantly in children, have been associated with hemopericardium (13), but the majority of viral infections result in inflammatory effusions without bleeding. Finally, in several instances, when no specific agent is found, the condition is termed idiopathic, although it may be related to inflammatory conditions secondary to undetected (viral) infections and autoimmune diseases (14).

## 4 Conclusion

Acute non-traumatic hemopericardium complicating acute pericarditis is a rare condition mostly due to neoplasm or tuberculous pericarditis. The patient`s clinical and epidemiological history suggested those pathologies as responsible for the disease, but ultimately was classified as idiopathic, likely secondary to viral infection. The case emphasizes the importance of history, laboratory tests and multimodal images for the accurate diagnosis and treatment of the disease.

## Data Availability

The original contributions presented in the study are included in the article/Supplementary Material, further inquiries can be directed to the corresponding author.
