# Supplementary figures and images for: Case Report: Spontaneous acute hemopericardium
Source: Front Cardiovasc Med. 2024 Oct 1;11:1414519. doi: 10.3389/fcvm.2024.1414519 (PMC11473430; doi:10.3389/fcvm.2024.1414519)

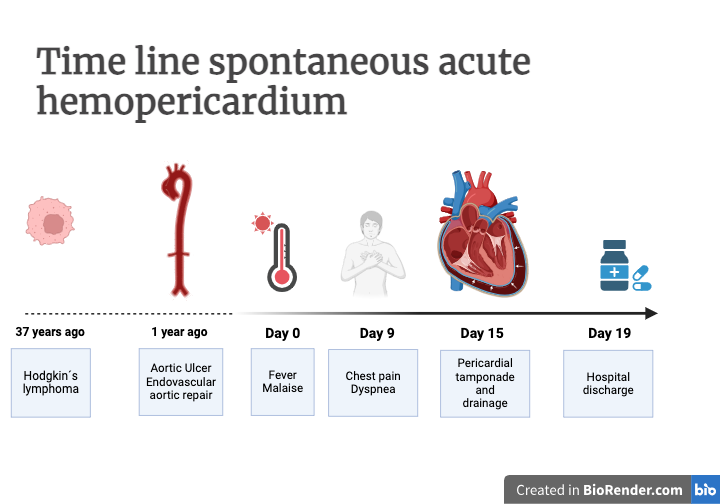

Supplement: Supplementary file 2 [file Image1.png]
